# Advancing the Therapeutic Frontier in Thyroid Eye Disease

**DOI:** 10.17925/EE.2025.21.2.2

**Published:** 2025-06-30

**Authors:** Seyed Omid Mohammadi, Coby Ray

**Affiliations:** 1. School of Medicine, Texas Christian University, Fort Worth, TX, USA; 2. Ophthalmology, Texas Tech University Health Sciences Center, Lubbock, TX, USA

**Keywords:** Bactoclimab, exophthalmos, Graves' ophthalmopathy, hyperthyroidism, interleukin-6, teprotumumab, thyroid eye disease, tumour necrosis factor-alpha

## Abstract

Thyroid eye disease (TED), also known as Graves’ orbitopathy, is driven by autoimmune processes that lead to orbital inflammation, oedema and potential vision loss. Traditional management includes high-dose intravenous corticosteroids, orbital radiotherapy and surgery. However, newer therapies, notably teprotumumab (a monoclonal antibody against the insulin-like growth factor I receptor), offer targeted immunomodulation with promising outcomes in reducing proptosis and improving symptoms. The future of TED treatment lies in further research on biologics, personalized approaches and continued multidisciplinary collaboration to optimize patient care.

Thyroid eye disease (TED), also known as Graves’ orbitopathy, is a complex autoimmune disorder driven by an interplay of immune cells, orbital fibroblasts and tissue remodelling factors that lead to inflammation, oedema and, ultimately, potential vision loss.^1^ While the disease has historically been challenging to manage, recent therapeutic innovations are reshaping treatment paradigms and offering new hope for patients. This editorial focuses on reviewing recent advances in the management of TED.

## Pathophysiology and clinical burden

The pathogenesis of TED primarily involves autoantibodies directed against the thyroid-stimulating hormone receptor and, in many cases, the insulin-like growth factor I receptor (IGF-1R).^2,3^ This autoimmune assault triggers the infiltration of T cells, B cells and macrophages into the orbital tissues. When activated, orbital fibroblasts produce glycosaminoglycans, expanding orbital fat and extraocular muscles. The consequent inflammatory and volumetric changes manifest as proptosis, eyelid retraction, diplopia and, in severe cases, compressive optic neuropathy.^2^

TED imposes a significant physical and psychosocial burden on affected individuals. Progressive disease has the potential to compromise ocular function and cause pain, tearing and photophobia. Additionally, disfiguring proptosis can negatively impact self-esteem and quality of life.^4^ Given these multifaceted challenges, an effective treatment strategy for TED addresses not only the acute inflammatory state but also the chronic fibrotic components and the patient’s overall wellbeing.

## Established treatments: Corticosteroids and more

Besides general measures recommended for all patients (smoking cessation, hypercholesterolemia, etc.) traditionally, moderate-to-severe active TED has been treated with high-dose intravenous glucocorticoids.^5^ When administered appropriately, corticosteroids can mitigate acute inflammation and slow disease progression. However, their broad immunosuppressive effects and potential adverse events, such as hyperglycaemia, cardiovascular complications and hepatic toxicity, limit long-term use.

Other conventional modalities include the following:

- **Orbital radiotherapy:** low-dose orbital radiotherapy has been used to modulate the inflammatory process. Although it can provide relief in some patients, its efficacy is variable, and concerns about radiation-induced side effects limit more widespread use.
- **Surgical interventions:** orbital decompression, strabismus surgery and eyelid procedures remain pivotal when disease activity plateaus or in urgent situations such as compressive optic neuropathy. While advances in surgical techniques, including endoscopic approaches, have reduced morbidity and improved cosmetic outcomes, surgery often addresses the consequences of tissue expansion and fibrosis rather than addressing the underlying disease process.

### Novel therapies: Targeted biologics

Recent research has focused on immunomodulatory therapies targeting the molecular drivers of TED. These novel agents aim to inhibit specific pathways implicated in orbital tissue inflammation, offering potentially greater efficacy and a more favourable safety profile than glucocorticoids.

#### Teprotumumab

A significant advance in TED management is teprotumumab, a human monoclonal antibody that inhibits IGF-1R. By blocking the IGF-1R signalling cascade, teprotumumab effectively reduces inflammation and glycosaminoglycan deposition within the orbit. Clinical trials have demonstrated its efficacy in reducing proptosis, improving ocular symptoms (pain, swelling and diplopia) and enhancing the quality of life by alleviating disfigurement and providing symptomatic relief.^1^

While teprotumumab has been acknowledged as a therapeutic milestone, there are still questions regarding its long-term safety, optimal timing for initiation and potential benefits in later fibrotic stages of the disease. Ongoing post-marketing surveillance and real-world evidence are needed to provide clarity on its safety profile and sustained efficacy.

#### Batoclimab

Batoclimab is a monoclonal antibody targeting the neonatal fragment crystallizable receptor (FcRn) that has shown promising potential in managing TED. Through inhibiting FcRn, batoclimab accelerates the degradation of circulating pathogenic IgG antibodies, particularly thyroid-stimulating immunoglobulins, which are central to TED pathogenesis.^6^ A study by Lee and Kahaly presented batoclimab as an emerging targeted therapy capable of modulating disease activity by dampening thyroid-stimulating hormone receptor-driven immune responses. Although TED-specific clinical trials for batoclimab are yet to be published, its successful use in other autoimmune diseases, such as myasthenia gravis, reinforces its viability as a therapeutic candidate.^7^

Compared with other biologics such as teprotumumab, batoclimab offers a broader approach by lowering all IgG subclasses, thereby potentially affecting multiple immune pathways contributing to TED. The drug’s systemic immunomodulatory effects could offer an advantage in patients with overlapping autoimmune conditions or those intolerant to more narrowly acting agents, such as teprotumumab. The current studies underscore the importance of batoclimab’s favourable safety profile and its capacity to rapidly reduce pathogenic antibody levels without causing widespread immunosuppression. This makes it an attractive option for long-term disease control. However, more thorough phase II or III trials focused specifically on TED are necessary to confirm batoclimab’s clinical utility, including endpoints such as proptosis reduction, improvement in clinical activity score and enhancements in vision-related quality of life. It is important to note that there is a report of a double-blind randomized controlled trial assessing the 12-week change in proptosis in patients with active moderate-to-severe TED. However, the trial was prematurely terminated due to an unexpected increase in serum cholesterol levels in the treatment group. Despite this, the study demonstrated good tolerability of batoclimab, with the reversal of drug-induced hypercholesterolemia and hypoalbuminemia within 8 weeks of discontinuation.^8^ As our understanding of TED’s immunopathology deepens, targeting FcRn for immunomodulation may emerge as a cornerstone in the next generation of TED therapeutics.

## Emerging biologic agents

Beyond IGF-1R inhibition, several other biological agents, including interleukin-6 (IL-6) inhibitors, tumour necrosis factor-alpha (TNF-α) inhibitors and rituximab, are under investigation.

- **IL-6 inhibitors**: given the role of cytokines such as IL-6 in propagating orbital inflammation, blockade of IL-6 signalling may help attenuate disease activity.^9^
- **TNF-α inhibitors**: in certain autoimmune disorders, TNF-α drives tissue damage. While TNF-α inhibitors have shown promise in a range of inflammatory conditions, their precise role in TED remains to be fully defined.^10^
- **Rituximab**: by depleting CD20+ B cells, rituximab has shown variable efficacy in TED, but further research is needed to delineate which subgroups of patients might benefit most.^11^

These novel agents highlight the growing emphasis on targeted immunomodulation, with an overarching goal of improving outcomes while limiting off-target side effect**s**.

## Multidisciplinary care

Optimal care for patients with TED requires close collaboration among ophthalmologists, endocrinologists and other healthcare professionals. To best serve the patients, some considerations include early diagnosis, risk stratification, monitoring and long-term follow-ups:

- **Early diagnosis and risk stratification**: identifying patients at risk for severe disease (e.g. those with high baseline inflammation, active smoking status or uncontrolled thyroid dysfunction) enables earlier intervention. Additionally, as immunomodulatory therapies expand, clinicians must individualize therapy based on clinical severity, disease phase (active versus fibrotic) and comorbidities.
- **Monitoring and long-term follow-up**: regular ophthalmic examinations addressing proptosis measurements, extraocular muscle function and optic nerve assessment remain essential in treating patients suffering from TED. Continued imaging can help detect early signs of optic nerve compression. Additionally, given the psychosocial impact of TED, supportive measures such as vision rehabilitation, counselling and smoking cessation programs should also have a role in the comprehensive management of patients with TED.^12^

### Future directions and conclusions

The therapeutic landscape of TED is evolving rapidly, steered by better insight into autoimmune pathways and the development of precision biologics. Although current data strongly support the efficacy of teprotumumab, it is crucial that the medical community continues to refine patient selection criteria and explore additional agents that may offer complementary or synergistic benefits.^1^

Given the current understanding of TED treatments, priorities moving forward include extended follow-up studies to define the durability of response and late treatment-related events and evaluate outcomes of combining different targeted agents or adding short courses of corticosteroids during acute phases. Another critical area is identifying molecular markers to predict therapeutic response and guide the choice of biologics for personalized management. It is important to mention that even with successful medical therapy, a subset of patients will inevitably require surgical intervention and advances in minimally invasive techniques and computer-assisted navigation hold great promise for improving both functional and cosmetic outcomes.

In conclusion, targeted and mechanism-based interventions for TED have arrived. For clinicians and patients alike, these developments signal a transformative shift in how TED can be managed, moving from broad immunosuppression to precision medicine grounded in a detailed understanding of disease pathophysiology. As research continues to clarify novel pathways and refine existing therapies, the global ophthalmic community can anticipate progressing strategies that preserve sight and restore quality of life for individuals afflicted by TED.

## Summary

TED, also known as Graves’ orbitopathy, is driven by autoimmune processes that lead to orbital inflammation, oedema and potential vision loss. Traditional management includes high-dose intravenous corticosteroids, orbital radiotherapy and surgery. However, newer therapies, notably teprotumumab (a monoclonal antibody against the insulin-like growth factor I receptor) and batoclimab, offer targeted immunomodulation with promising outcomes in reducing proptosis and improving symptoms. The future of TED treatment lies in further research on biologics, personalized approaches and continued multidisciplinary collaboration.

## References

[1] Householder NA, Ray C (2024). Teprotumumab’s impact on proptosis in long-duration thyroid eye disease: A systematic review and meta-analysis.. touchREV Endocrinol..

[2] Smith TJ, Kahaly GJ, Ezra DG (2017). Teprotumumab for thyroid-associated ophthalmopathy.. N Engl J Med..

[3] Moledina M, Damato EM, Lee V (2024). The changing landscape of thyroid eye disease: Current clinical advances and future outlook.. Eye..

[4] Wickwar S, McBain HB, Ezra DG (2014). What are the psychosocial outcomes of treatment for thyroid eye disease? A systematic review.. Thyroid..

[5] Bartalena L, Kahaly GJ, Baldeschi L (2021). The 2021 European Group on Graves’ orbitopathy (EUGOGO) clinical practice guidelines for the medical management of Graves’ orbitopathy.. Eur J Endocrinol..

[6] Gjølberg TT, Mester S, Calamera G (2025). Targeting the neonatal FC receptor in autoimmune diseases: Pipeline and progress.. BioDrugs..

[7] Lee ACH, Kahaly GJ (2025). Targeted immunotherapies for Graves’ thyroidal & orbital diseases.. Front Immunol..

[8] Yoon JS, Kikkawa DO (2022). Thyroid eye disease: From pathogenesis to targeted therapies.. Taiwan J Ophthalmol..

[9] Perez-Moreiras JV, Gomez-Reino JJ, Maneiro JR (2018). Efficacy of tocilizumab in patients with moderate-to-severe corticosteroid-resistant graves orbitopathy: A randomized clinical trial.. Am J Ophthalmol..

[10] Ferreira GA, Teixeira AL, Calderaro DC (2016). Atorvastatin reduced soluble receptors of TNF-alpha in systemic lupus erythematosus.. Clin Exp Rheumatol..

[11] Salvi M, Vannucchi G, Currò N (2015). Efficacy of B-cell targeted therapy with rituximab in patients with active moderate to severe Graves’ orbitopathy: A randomized controlled study.. J Clin Endocrinol Metab..

[12] Vestergaard P (2002). Smoking and thyroid disorders – a metaanalysis.. Eur J Endocrinol..

